# Synthesis of Sea Urchin-Like NiCo_2_O_4_ via Charge-Driven Self-Assembly Strategy for High-Performance Lithium-Ion Batteries

**DOI:** 10.1186/s11671-018-2819-4

**Published:** 2019-01-07

**Authors:** Bin Wang, Chi-Wing Tsang, Ka Ho Li, Yuanyuan Tang, Yanping Mao, Xiao-Ying Lu

**Affiliations:** 1 Faculty of Science and Technology, Technological and Higher Education Institute of Hong Kong, Hong Kong, People’s Republic of China; 2 Hong Kong Applied Science and Technology Research Institute, Hong Kong, People’s Republic of China; 3 School of Environmental Science and Engineering, Southern University of Science and Technology, Shenzhen, People’s Republic of China; 4 Department of Chemistry and Environmental Engineering, Shenzhen University, Shenzhen, People’s Republic of China

**Keywords:** Hydrothermal synthesis, NiCo_2_O_4_, Self-assembly, Lithium-ion batteries

## Abstract

In this study, hydrothermal synthesis of sea urchin-like NiCo_2_O_4_ was successfully demonstrated by a versatile charge-driven self-assembly strategy using positively charged poly(diallydimethylammonium chloride) (PDDA) molecules. Physical characterizations implied that sea urchin-like microspheres of ~ 2.5 μm in size were formed by self-assembly of numerous nanoneedles with a typical dimension of ~ 100 nm in diameter. Electrochemical performance study confirmed that sea urchin-like NiCo_2_O_4_ exhibited high reversible capacity of 663 mAh g^−1^ after 100 cycles at current density of 100 mA g^−1^. Rate capability indicated that average capacities of 1085, 1048, 926, 642, 261, and 86 mAh g^−1^ could be achieved at 100, 200, 500, 1000, 2000, and 3000 mA g^−1^, respectively. The excellent electrochemical performances were ascribed to the unique micro/nanostructure of sea urchin-like NiCo_2_O_4_, tailored by positively charged PDDA molecules. The proposed strategy has great potentials in the development of binary transition metal oxides with micro/nanostructures for electrochemical energy storage applications.

## Introduction

Spinel nickel cobaltite (NiCo_2_O_4_) is one of the most important binary transition metal oxides (TMOs) with wide applications in electro-catalytic water splitting, supercapacitors and rechargeable battery materials, etc. [1–7]. Particularly, spinel NiCo_2_O_4_, having a theoretical specific capacity (890 mAh g^−1^), can be used as promising high-capacity anode materials for electrochemical lithium storage, owing to the higher electrical conductivity and electrochemical activities than monometallic oxides (Co_3_O_4_ and NiO) [8, 9]. However, lithium storage performance of NiCo_2_O_4_ was highly dependent on the distinct structure and morphology, which showed significant effects on cycling stability and rate capability.

In recent years, various NiCo_2_O_4_ with interesting morphologies, including nanowires [10], nanosheets [11], nanoflakes [12], nanobelts [12], sea urchin-like [13], and flower-like structures [14], have been synthesized by hydrothermal and solvothermal method. Previous studies suggested that micro/nanostructures manifested dual benefits from microscale and nanoscale dimensions for improved electron and ion transport, thereby leading to superior electrochemical performances [15, 16]. Generally, structure design of NiCo_2_O_4_ with micro/nanostructures was directed by choosing appropriate morphology controlling reagents. Zhang et al. employed polyvinylpyrrolidone (PVP) to synthesize NiCo_2_O_4_ for controlling morphology, based on coordination of metal ions with functional groups (e.g., -N and/or C=O) of pyrrolidone [17]. However, limited effective structure directing reagents are feasible for synthesis of binary TMOs with unique morphology. Thus, it is highly desirable to explore versatile reagents for synthesizing NiCo_2_O_4_ with micro/nanostructures. Recently, we reported positively charged reagents, such as diallyldimethylammonium chloride (DDA) and its homopolymer, exhibited potentials in synthesizing Co_3_O_4_ for lithium-ion batteries (LIBs) [15, 16]. However, we are not aware of any binary TMOs (e.g., NiCo_2_O_4_) with micro/nanostructures synthesized by such charged molecules for electrochemical lithium storage applications.

Herein, we reported charge-driven self-assembly strategy for NiCo_2_O_4_ with sea urchin-like structure, followed by thermal treatment. The positively charged poly(diallydimethylammonium chloride) (PDDA) molecules were considered as a crucial structure directing reagent in hydrothermal synthesis. Sea urchin-like NiCo_2_O_4_ with micro/nanostructures also demonstrated superior lithium storage performance in repeated charge-discharge cycles. Obviously, it is the first work on charge-driven self-assembly synthesis of binary TMOs with assistance of charged organic molecules. This novel strategy is expected to pave a new way of synthesizing binary TMOs with novel micro/nanostructures for energy storage materials.

## Methods

### Synthesis of Sea Urchin-Like NiCo_2_O_4_

In a typical synthesis, 0.5 g of nickel acetate tetrahydrate (≥ 99%), 1.0 g of cobalt acetate tetrahydrate (≥ 98%), and 3.0 g of urea (99.5%) received from Acros Organics were dissolved in 55 mL deionized water, followed by adding 5 g PDDA solution (20 wt.% in H_2_O, Sigma-Aldrich). The mixed solution was carefully transferred into a sealed Teflon-lined stainless steel autoclave and placed in an electric oven maintained at 120 °C for 2 h. The resulting precipitation was collected by vacuum-assisted filtration and washed with deionized water for three times. Finally, the filtered sample was thermal treated in a muffle furnace at 450 °C for 2 h. The as-synthesized black samples were directly used in material characterizations and electrochemical performance evaluation.

### Material Characterizations and Electrochemical Performance Evaluation

Crystal phases, material morphologies, microstructures, and valence states of the as-prepared samples were characterized by powder X-ray diffractometer (XRD, Philips PW1830), field emission scanning electron microscope (FE-SEM, Hitachi S4800), transmission electron microscope (TEM, FEI Tecnai G^2^ 20 scanning), and X-ray photoelectron spectroscopy (XPS, Model PHI5600), respectively. Thermal conversion study of precursors was conducted on thermogravimetric analysis (TGA, Mettler Toledo) and differential scanning calorimetry (DSC, Mettler Toledo) under oxygen atmosphere. In addition, specific surface area and pore size distributions of NiCo_2_O_4_ were performed on a surface area analyzer (Quantachrome Instruments) by N_2_ adsorption-desorption isotherms at 77 K. The specific surface area and pore size distribution were obtained by multi-point Brunauer–Emmett–Teller (BET) and Barrett–Joyner–Halenda (BJH) method, respectively. Electrochemical lithium storage performance and rate capability were evaluated in CR2025 coin-type cell with NiCo_2_O_4_ as working electrode, lithium metal as counter electrode, microporous membrane (Celgard® 2400) as separator, and 1 M LiPF_6_ in 50 vol.% ethylene carbonate and 50 vol.% dimethyl carbonate as electrolyte. The working electrode was composed of 80% active NiCo_2_O_4_ materials, 10% PVdF binder, and 10% SuperP conductive carbon. Cyclic voltammetry (CV) analysis was measured in the voltage range of 0.005–3 V vs. Li^+^/Li and electrochemical impedance spectra (EIS) of sea urchin-like NiCo_2_O_4_ anodes were also recorded on electrochemical station (CorrTest® Instruments) in the frequency range of 100 kHz to 0.01 Hz with an amplitude of 5 mV. Galvanostatic charge-discharge test was conducted on a battery testing system (LAND CT2001A) at room temperature. The cycling performance was conducted at a current density of 100 mA g^−1^ for 100 cycles and rate capability test was performed with various current densities ranging from 100 mA g^−1^ to 3000 mA g^−1^.

## Results and Discussion

**Fig. 1:**
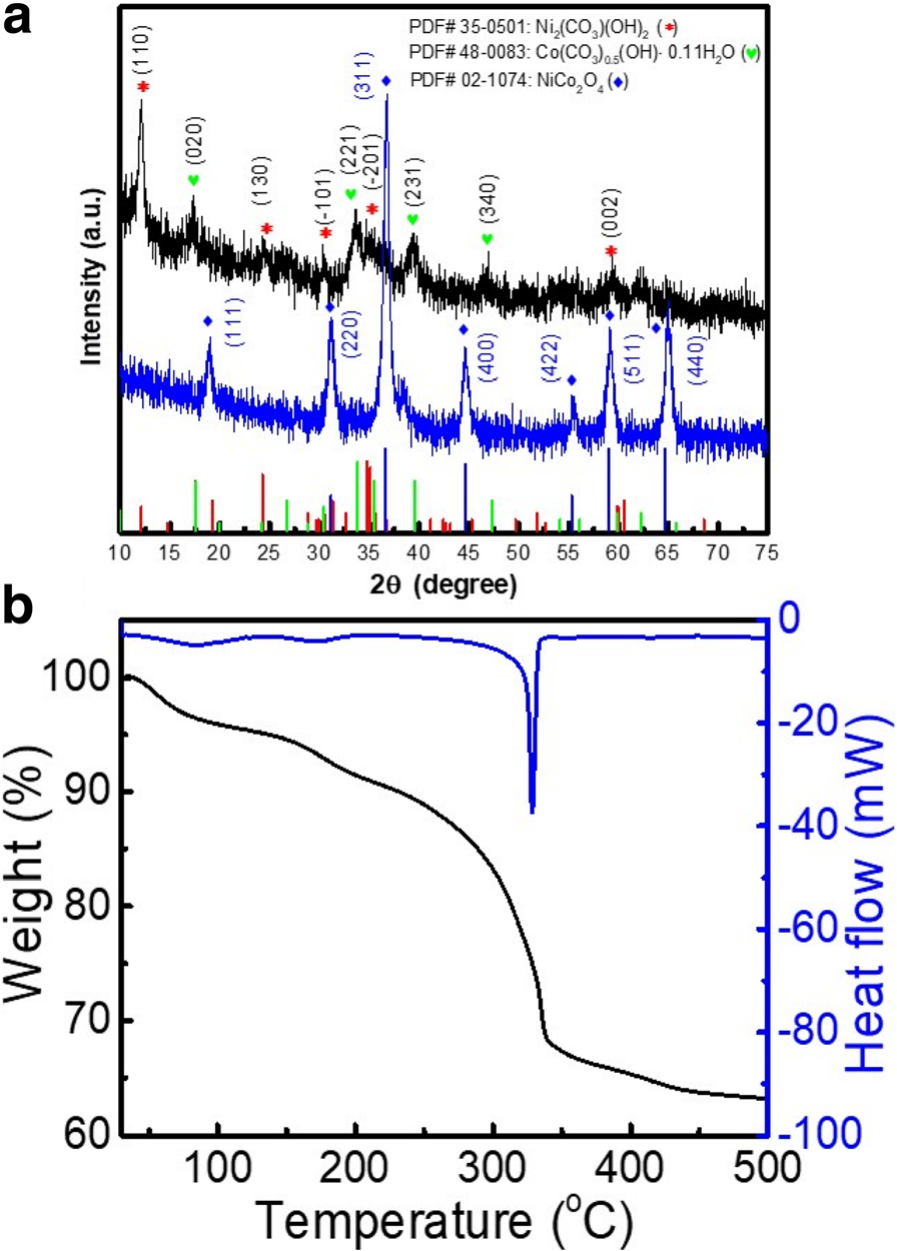
**a** XRD patterns of the as-prepared precursor and NiCo_2_O_4_ product before and after heat treatment at 450 °C. **b** TGA analysis of precursor under oxygen atmosphere with a heating rate of 10 °C min^−1^

XRD pattern in Fig. 1a suggested that the as-prepared product was face-centered-cubic NiCo_2_O_4_ of high crystallinity and purity (PDF 02-1074). The 2θ peaks located at 31.1°, 36.6°, 44.6°, 55.3°, 59.0°, 64.7° were assigned to characteristic crystal planes (2 2 0), (3 1 1), (4 0 0), (4 2 2), (5 1 1), and (4 4 0), respectively. Moreover, crystal phases in the as-prepared precursors were consisted of Ni_2_CO_3_(OH)_2_ (PDF 35-0501), and Co(CO_3_)_0.5_(OH)·0.11H_2_O (PDF 48-0083), consistent with previous study [18]. The 2θ peaks at 12.1°, 24.3°, 30.5°, 34.8°, and 59.8° could be related to Ni_2_CO_3_(OH)_2_ crystal plane (1 1 0), (1 3 0), (− 1 0 1), (− 2 0 1), and (0 0 2) respectively. The 2θ peaks at 17.5°, 33.8°, 39.5°, and 47.3° could be attributed to Co(CO_3_)_0.5_(OH)·0.11H_2_O crystal plane (0 2 0), (2 2 1), (2 3 1), and (3 4 0), respectively. Apparently, both Ni^2+^ and Co^2+^ were precipitated by CO_3_^2−^ and OH^−^ ions, released from the decomposition of urea at hydrothermal conditions [16]. TGA curve in Fig. 1b displayed that calcination temperature of 450 °C was enough for thermal conversion of the mixed phases to pure NiCo_2_O_4_, since no mass loss was observed after 450 °C. Also, the conversion temperature was determined to be 350 °C, leading to a total mass loss of 37 wt %.

**Fig. 2:**
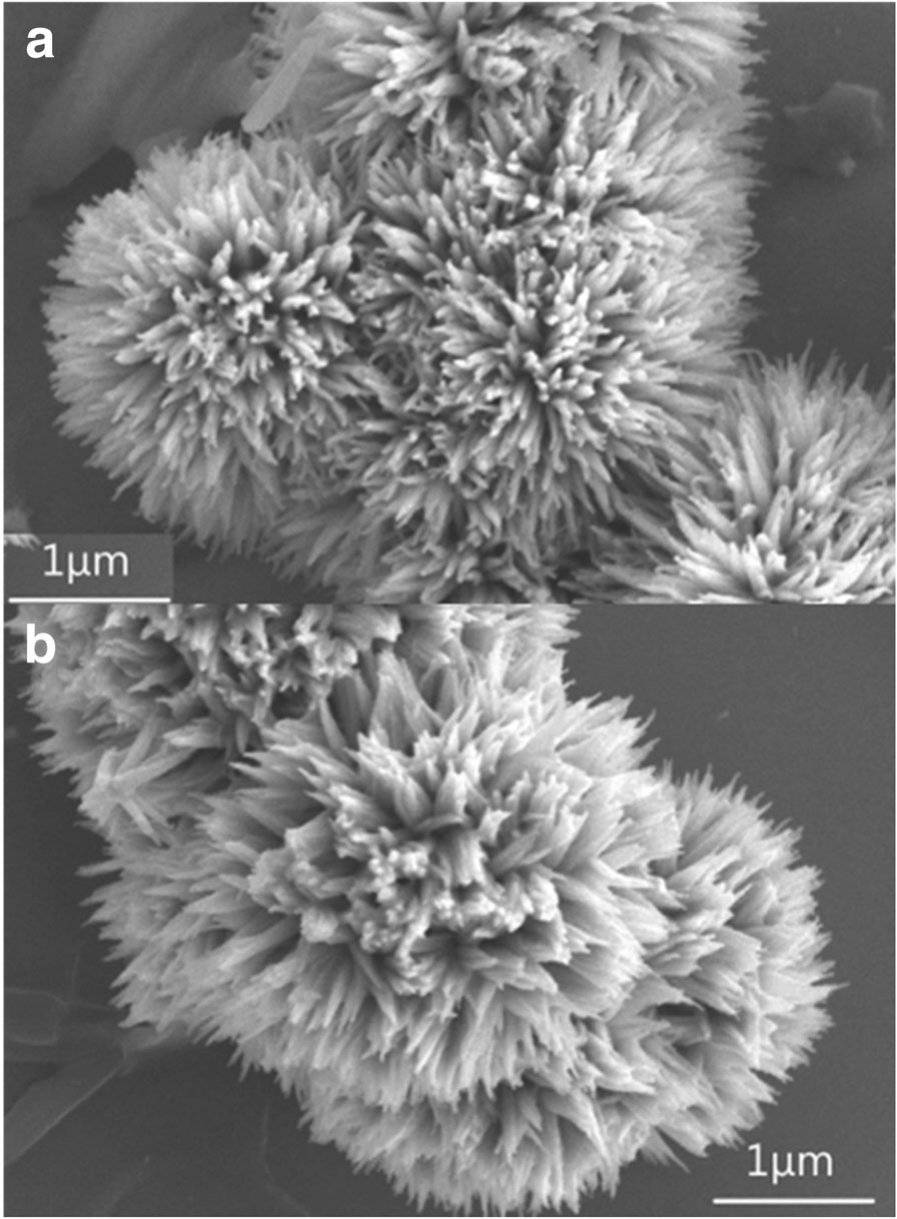
**a**, **b** Typical FE-SEM images of the sea urchin-like precursor and NiCo_2_O_4_ synthesized with 5 g PDDA solution

Morphological analysis in Fig. 2a, b implied that sea urchin-like structure of precursors was successfully obtained with PDDA-assisted hydrothermal treatment. After thermal treatment at 450 °C, sea urchin-like morphology of NiCo_2_O_4_ microspheres could still be maintained, indicating the robust nature at high temperature. The NiCo_2_O_4_ microspheres were typically ~ 2.5 μm in diameter, composed of numerous nanoneedles with an average diameter of ~ 100 nm. Note that PDDA molecules play a pivotal role in the formation of sea urchin-like structure. At the beginning, the decomposition of urea leading to generation of CO_3_^2−^ and OH^−^ initiated the nucleation of Co^2+^ and Ni^2+^ at hydrothermal conditions. The nitrogen atoms in PDDA endowed with lone electron pairs enabled strong electrostatic interaction with negative ions. Therefore, the surface of these small nuclei was first occupied by these negative ions (CO_3_^2−^ and OH^−^), leading to electrostatic adsorption of positively molecules. Owing to steric hindrance, PDDA led to the crystal growth of precursors along a preferential direction. In order to minimize surface energy, self-assembly of nanostructures via a spontaneous Ostwald ripening process eventually occur, resulting in the formation of sea-urchin like structure.

**Fig. 3:**
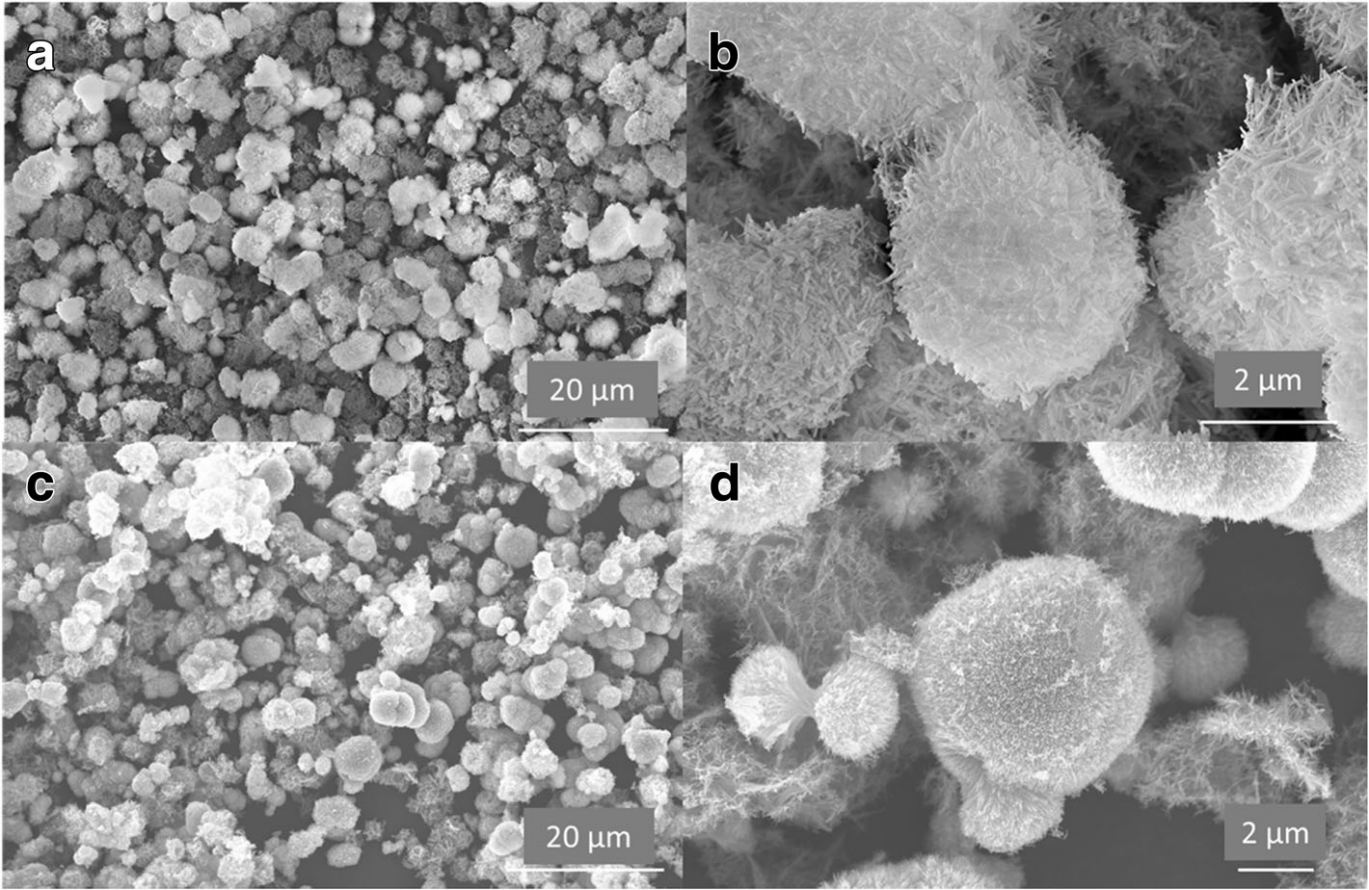
Typical FE-SEM images of the as-prepared precursor synthesized with different amounts of PDDA solution **a, b** 2.5 g; **c, d** 10 g

The effects of PDDA amounts on the morphology of precursors were also investigated with FE-SEM characterization. As shown in Fig. 3, when PDDA solution of 2.5 g was added in the hydrothermal synthesis, the as-prepared precursor sample exhibited the same spherical structure of 2~5 um in diameter. Many nanoneedles, considered as the building units, were randomly organized into the large micro/nanostructured spheres. When PDDA amount was further increased to 10 g, both sea urchin-like and straw-sheaf-like structures could be obviously found in the hydrothermal precursors. The effects of PDDA on crystal orientation should be associated with the surface charge property of small nuclei, which could be tailored by the amounts of positively charged PDDA molecules. Thus, PDDA solution of 5 g, which was equivalent to a concentration of 16.7 mg L^−1^, was the optimal conditions for synthesizing sea urchin-like structure, owing to the preferential crystal growth orientation.

**Fig. 4:**
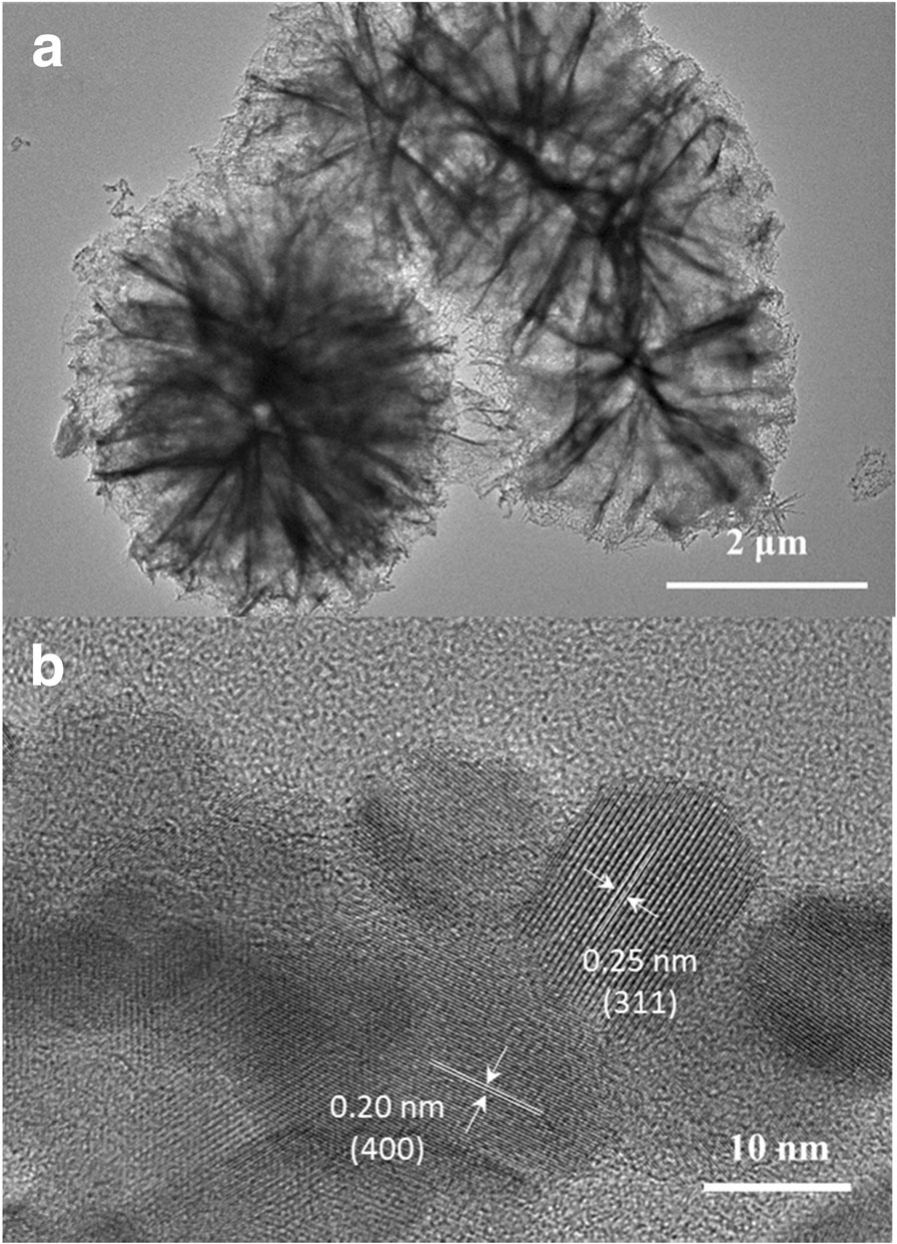
**a**, **b** TEM images of the sea urchin-like NiCo_2_O_4_ synthesized with 5 g PDDA solution

The microstructures of microspheres analyzed by TEM revealed that highly porous structures in NiCo_2_O_4_ was indicated by the evident white/black contrast and high crystallinity was convinced by the clear lattice planes (Fig. 4a, b). The average size of primary particles was about 10 nm. The *d*-spacing values of ~ 0.20 nm and ~ 0.25 nm were ascribed to crystal plane (400) and (311), respectively. In addition, the pore size was about 10 nm on average. The above analysis confirmed that sea urchin-like NiCo_2_O_4_ were successfully synthesized by charge-driven self-assembly strategy with subsequent thermal treatment.

**Fig. 5:**
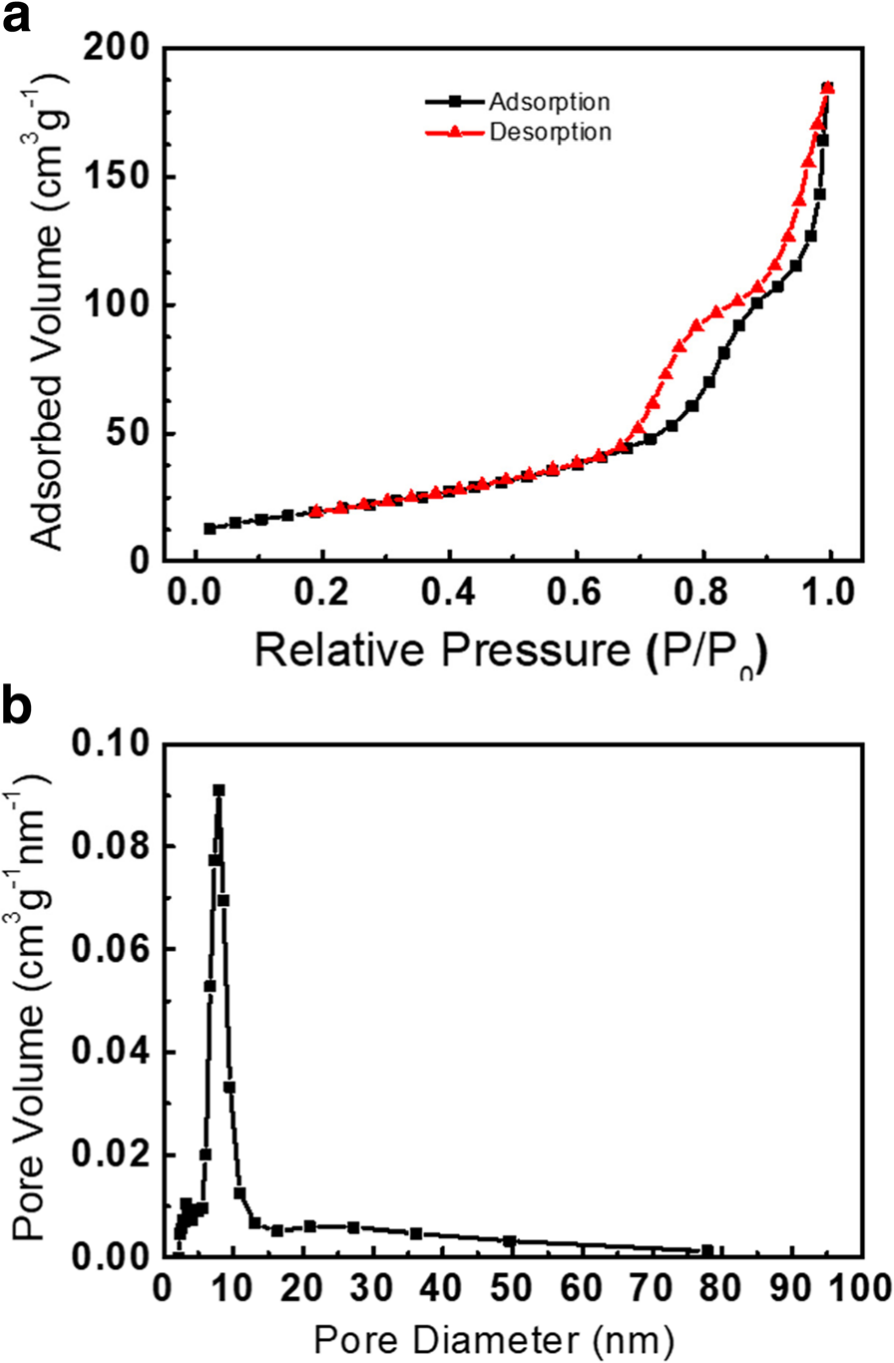
**a** Nitrogen adsorption and desorption isotherms and **b** pore size distribution of sea urchin-like NiCo_2_O_4_ synthesized with 5 g PDDA solution

**Fig. 6:**
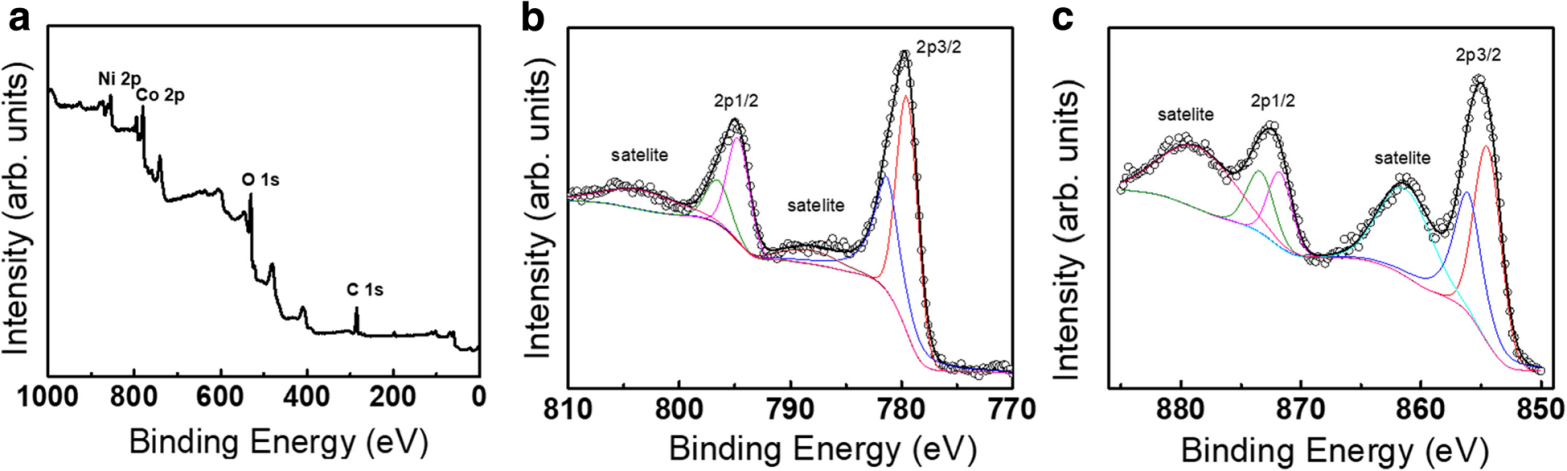
**a** Survey spectrum of sea urchin-like NiCo_2_O_4_. **b**, **c** High-resolution XPS spectra of Co2p and Ni2p

Based on N_2_ adsorption-desorption isotherm, BET-specific surface area and BJH pore size distribution of NiCo_2_O_4_ sample were about 68.6 m^2^ g^−1^ and 8.8 nm, respectively (Fig. 5). The high surface area and uniform pore size were favorable for shortening ion diffusion length and alleviating volume expansion in electrochemical processes. The survey spectrum in Fig. 6a depicted the presence of Ni, Co, O, and C in the product. The high-resolution XPS data of Co2p in Fig. 6b indicated that co-existence of Co^2+^ and Co^3+^ species, as revealed by the fitting Co2p_3/2_ peaks located at ~ 779.5 eV and ~ 781.3 eV, respectively. Similarly, high-resolution XPS data of Ni 2p in Fig. 6c implied the presence of Ni^2+^ and Ni^3+^, as suggested by the fitting Ni2p_3/2_ peaks centered at about ~ 854.6 eV and ~ 856.2 eV, respectively. The presence of satellite peaks also confirmed the presence of Co^2+^ and Ni^2+^. Note that the peak separations for Co2p_1/2_ vs Co2p_3/2_ and Ni2p_1/2_ vs Ni2p_3/2_ were determined to be 15.2 and 17.3 eV, consistent with previous studies [16, 19]. Multiple valence states of Co (+ 2, + 3) and Ni (+ 2, + 3) in spinel NiCo_2_O_4_ were beneficial for electrochemical conversion reactions in charging-discharging processes.

**Fig. 7:**
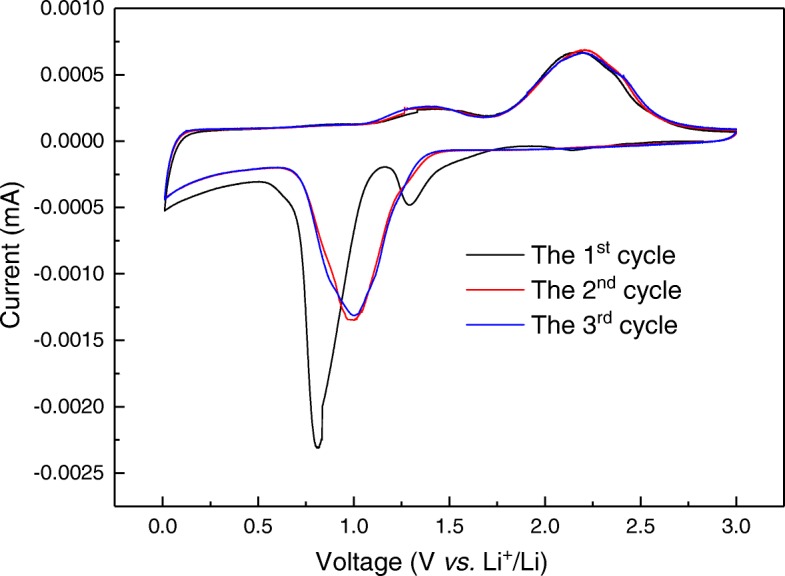
Cyclic voltammetry (CV) analysis of sea urchin-like NiCo_2_O_4_ anodes in the voltage range of 0.005–3.0 V with a scanning rate of 0.01 mV s^−1^

The electrochemical conversion mechanism and reversibility of sea urchin-like NiCo_2_O_4_ was investigated with CV analysis. As shown in Fig. 7, in the first cycle, two distinct cathodic peaks located at about 0.8 V and 1.3 V indicated the electrochemical reduction of Co^3+^ to Co^2+^, and then reduction of Co^2+^ and Ni^2+^ to metallic Co and Ni species, respectively [20]. For the first anodic process, electrochemical oxidation of metallic Co and Ni at about 1.4 and 2.2 V would lead to the reversible generation of Co^2+^, Co^3+^, and Ni^2+^ species, which eventually resulted in the formation of NiCo_2_O_4_ phase. It is also possible that solid electrolyte interphase was formed in the first activation cycle. Obviously, after the activation process in the first cycle, good reversibility of electrochemical redox reactions could be observed in the subsequent two cycles, as indicated by the overlapped CV curves. The only difference was that the major reduction peak was shifted from 0.8 to 1.0 V, consistent with previous CV study on NiCo_2_O_4_ anodes [8]. The detailed mechanism of electrochemical conversion reactions was also discussed in previous studies and could be described as below [20].

1 $$ {\mathrm{NiCo}}_2{\mathrm{O}}_4+8\ {\mathrm{Li}}^{+}+8\ {\mathrm{e}}^{\hbox{-}}\leftrightarrow 2\ \mathrm{Co}+\mathrm{Ni}+4\ {\mathrm{Li}}_2\mathrm{O} $$

2 $$ \mathrm{Ni}+{\mathrm{Li}}_2\mathrm{O}\leftrightarrow \mathrm{Ni}\mathrm{O}+2\ {\mathrm{Li}}^{+}+2\ {\mathrm{e}}^{\hbox{-} } $$

3 $$ \mathrm{Co}+{\mathrm{Li}}_2\mathrm{O}\leftrightarrow \mathrm{Co}\mathrm{O}+2\ {\mathrm{Li}}^{+}+2\ {\mathrm{e}}^{\hbox{-} } $$

4 $$ \mathrm{CoO}+1/3\ {\mathrm{Li}}_2\mathrm{O}\leftrightarrow 1/3\ {\mathrm{Co}}_3{\mathrm{O}}_4+2/3\ {\mathrm{Li}}^{+}+2/3\ {\mathrm{e}}^{\hbox{-} } $$

**Fig. 8:**
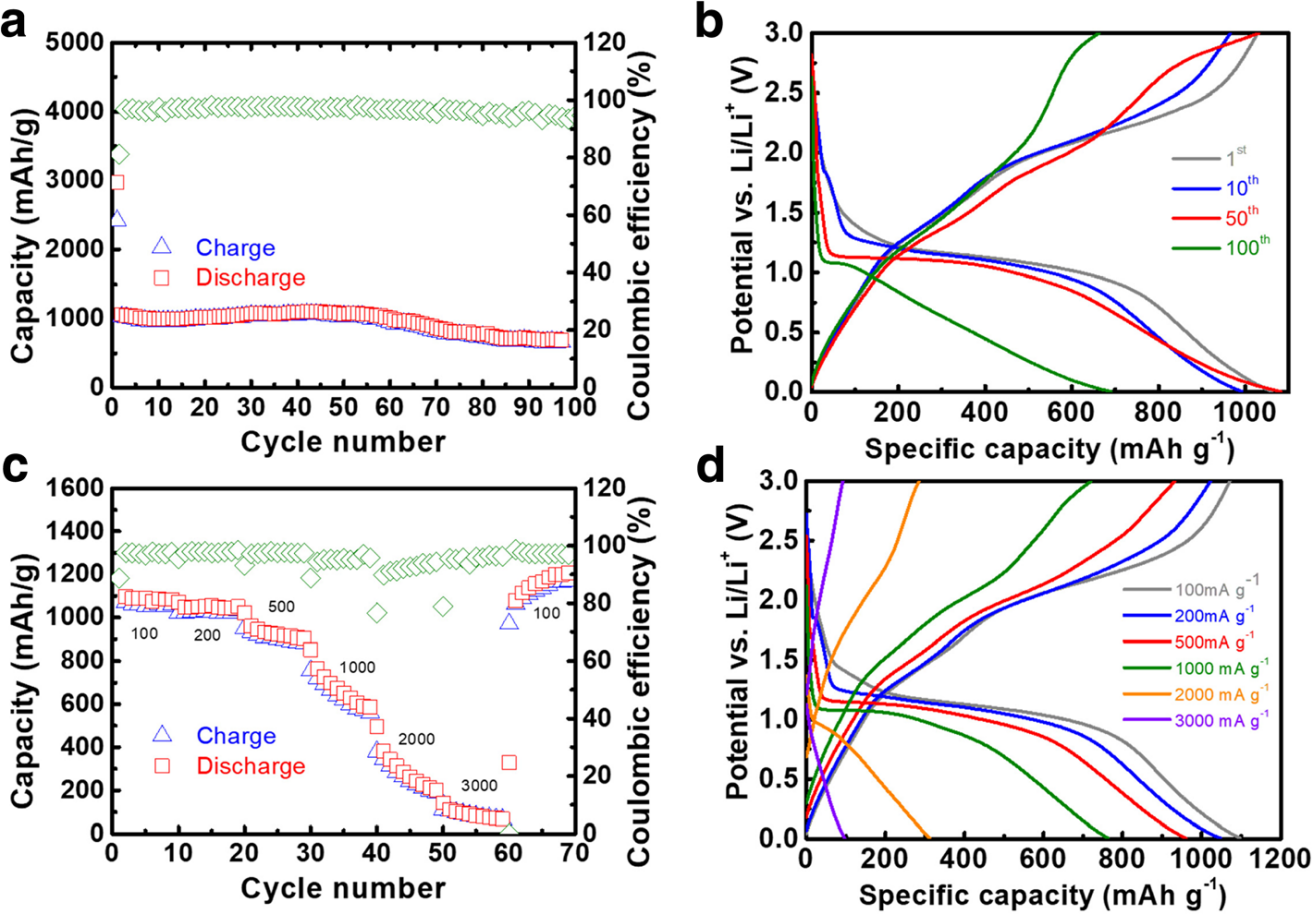
**a** Cycling performance of NiCo_2_O_4_ tested at a current density of 100 mA g^−1^. **b** Typical charge-discharge curves of NiCo_2_O_4_ tested at 100 mA g^−1^ for the 1st, 10th, 50th, and 100th cycle **c** rate capability performance. **d** Typical charge-discharge curves of NiCo_2_O_4_ tested at different current densities ranging from 100 to 3000 mA g^−1^

Electrochemical cycling performance of NiCo_2_O_4_ sample was provided in Fig. 8a and the result indicated that a reversible capacity of 663 mAh g^−1^ was achieved at a current density of 100 mA g^−1^ after 100 charge-discharge cycles. The cycling performance was also comparable with previous study on pure NiCo_2_O_4_ material. For example, electrochemical lithium storage of hierarchical NiCo_2_O_4_ nanowire array was about 413 mAh g^−1^ when evaluated at 100 mA g^−1^ over 100 cycles [5]. However, when NiCo_2_O_4_ was modified with highly conductive additives or metal oxides, better electrochemical performance could be achieved in comparison with pristine NiCo_2_O_4_. For instance, Chen et al. reported cycling stability of pure NiCoO_2_ was significantly improved by reduced graphene oxide and a high reversible capacity of 816 mAh g^−1^ was achieved with 80.1% capacity retention [21]. Also, Sun et al. reported the cycling performance of porous NiCoO_2_/NiO hollow dedecahedron was about 1535 mAh g^−1^ at 200 mA g^−1^ over 100 cycles, equivalent to a capacity retention of 97.2% [22]. The Coulombic efficiencies after the initial activation were almost stabilized at ~ 100%, indicative of high electrochemical reversibility. As shown in Fig. 8b, the charge-discharge curves at different cycles also showed distinctive behaviors. With repeated charge-discharge cycles, it is obvious that charge-discharge curves of the 50th cycle were also identical with the initial cycles, indicating similar electrochemical reaction pathways in the first 50 cycles. However, the charge-discharge curves of the 100th cycle showed slightly different behaviors, suggesting that slow material decay might be present during the anodic conversion reactions. Moreover, rate capability in Fig. 8c showed that the average discharge capacities of NiCo_2_O_4_ measured at current densities 100, 200, 500, 1000, 2000, and 3000 mA g^−1^ were about 1085, 1048, 926, 642, 261, and 86 mAh g^−1^, respectively. When current density was switched to 100 mA g^−1^, high reversible capacity of about 1000 mAh g^−1^ was still maintained, indicating no obvious decay of reversible capacity in rate capability test. Note that the experimental specific capacity of 1085 mAh g^−1^ achieved at 100 mA g^−1^ was higher than theoretical value (890 mAh g^−1^). This phenomenon was commonly observed in transition metal oxide anodes. The extra capacity might be ascribed to reversible formation of gel-like polymer films and interfacial lithium storage, etc. [23, 24]. In Fig. 8d, the typical charge-discharge curves at different current densities also suggested the specific capacity showed significant decrease with the increasing of charge-discharge current densities from 100 to 3000 mA g^−1^. The electrochemical performance achieved in this study was better or comparable with previous studies on NiCo_2_O_4_-based materials. For instance, Chen et al. reported mesoporous NiCo_2_O_4_ nanowires delivered reversible capacities of 1215, 797, and 413 mAh g^−1^ at current densities of 200, 500, and 1000 mA g^−1^, respectively [5]. The achieved rate capability of NiCo_2_O_4_ in this study was also comparable with previous work on other transition metal oxides. For example, Lyu et al. reported that reversible capacities of hollow CuO at evaluated current densities of 100, 200, 500, and 1000 mA g^−1^ were 629, 567, 488, and 421 mAh g^−1^, respectively [25]. It should be mentioned that the rate performance of sea urchin-like NiCo_2_O_4_ was not stable, particularly at high current densities. This phenomenon was probably due to semiconducting nature of pristine NiCoO_2_ and destruction of building units (nanoneedles) by high current density. Similarly, the C-rate performances of spherical NiCo_2_O_4_ and NiCo_2_O_4_ nanoribbons were also unstable in previous studies, when charge-discharge current density was changed to ≥ 1000 mA g^−1^ [20, 26].

Note that fluctuation of coulombic efficiency was also observed in the C-rate measurement, particularly at the changing points of current densities. For instance, when the current density was switched from 1000 to 2000 mA g^−1^, coulombic efficiency of the 40th cycle was suddenly declined from 100 to about 80%. In the following 9 cycles, coulombic efficiency was immediately stabilized at about 100%. The sudden drop of coulombic efficiency might be related to the partial loss of electrical connectivity between NiCo_2_O_4_ materials and conductive network by volume variation in the charging process, due to the applied high current density. Similar phenomena were also reported in previous C-rate studies on anode materials for rechargeable batteries [27, 28].

**Fig. 9:**
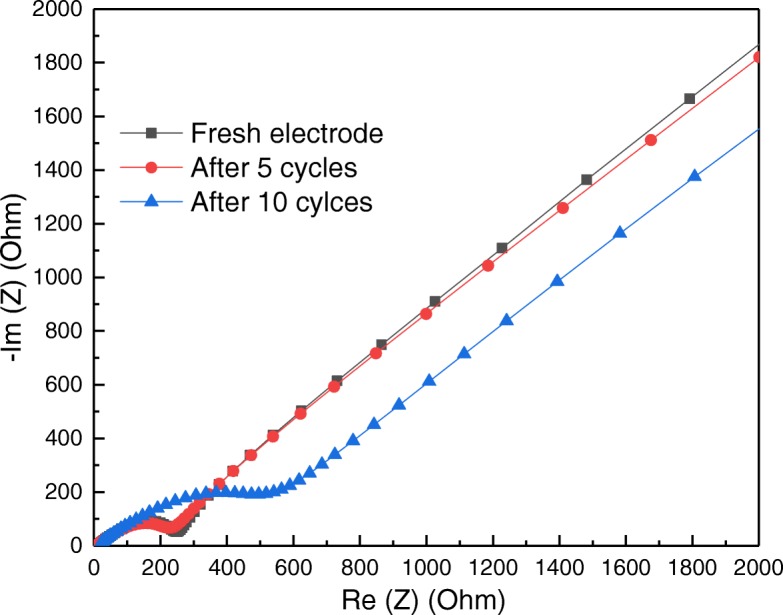
EIS spectra of sea urchin-like NiCo_2_O_4_ anodes after different cycling tests in a coin cell

To understand the nature of NiCo_2_O_4_ anodes, EIS analysis was conducted in the frequency range of 100 kHz to 0.01 Hz with an amplitude of 5 mV. EIS was widely employed as a useful tool to reveal electrochemical behaviors and charge transfer process [29, 30]. For NiCo_2_O_4_ anodes tested with different cycles, EIS spectra in Fig. 9 revealed small semicircles and straight lines in the high and low frequency regions, respectively. The small semicircles should be related to charge transfer resistance between electrode and electrolyte. The straight lines indicated the Warburg impedance, which should be associated with solid state diffusion of Li^+^ in NiCo_2_O_4_ electrodes [8]. The charge transfer resistances of fresh NiCo_2_O_4_ electrode before and after 5 cycles were almost identical, indicating no obvious change in electrode/electrolyte interface. However, after 10 cycles, charge transfer resistance became dominant in electrochemical processes, as indicated by a larger diameter of semicircle. In addition, the nearly parallel lines suggested the same solid-state Li^+^ diffusion behaviors before and after cycling tests. Therefore, charge transfer resistance of NiCo_2_O_4_ anodes could play a relatively important role in the electrochemical performance.

In this study, the improved performance of NiCo_2_O_4_ should be attributed to the micro/nanostructures of sea urchin-like morphology, compared to previous work on nanostructures (e.g., mesoporous nanowires). Basically, the lithium storage performance was associated with efficient transport of lithium ions and electrons in electrochemical charge-discharge cycles. The numerous nanoneedle, viewed as the building unit of sea urchin-like structure, could greatly improve solid-state Li^+^ diffusion behaviors, due to the shortened nanoscale length. In addition, the uniform microspheres, regarded as the secondary particles of sea urchin-like structure, could significantly enhance electron transport behaviors, owing to long-range electron transport network. The combined benefits of micro/nanostructures in sea urchin-like structure could result in better electrochemical performance than nanostructures. Overall, the superior electrochemical performance of NiCo_2_O_4_ was ascribed to the unique physical properties of sea urchin-like structure, which were tailored by PDDA-assisted charge-driven self-assembly strategy. This proposed strategy has potential in facile synthesis of energy storage materials for next generation LIBs.

## Conclusions

In conclusion, sea urchin-like NiCo_2_O_4_ were successfully synthesized by charge-driven self-assembly strategy with positively charged PDDA, followed by thermal treatment. The charged molecules play a pivotal role in the formation of sea urchin-like structure, due to electrostatic adsorption and steric hindrance. Also, sea urchin-like NiCo_2_O_4_ demonstrated great potentials in electrochemical lithium storage. The superior performance was ascribed to the unique sea urchin-like structure of NiCo_2_O_4_ for enhanced electron and ion transport_._ Overall, charge-driven self-assembly strategy is an appealing route to synthesize energy storage materials for high-performance lithium-ion batteries.

## Abbreviations

BET: Brunauer–Emmett–Teller
BJH: Barrett–Joyner–Halenda
CV: Cyclic voltammetry
DSC: Differential scanning calorimetry
EIS: Electrochemical impedance spectra
FE-SEM: Field emission scanning electron microscope
LIBs: Lithium-ion batteries
PDDA: Poly(diallydimethylammonium chloride)
TGA: Thermogravimetric analysis
TMOs: Transition metal oxides
XPS: X-ray photoelectron spectroscopy
XRD: X-ray diffractometer
